# Silicon amendment to rice plants impairs sucking behaviors and population growth in the phloem feeder *Nilaparvata lugens* (Hemiptera: Delphacidae)

**DOI:** 10.1038/s41598-017-01060-4

**Published:** 2017-04-24

**Authors:** Lang Yang, Yongqiang Han, Pei Li, Lizhang Wen, Maolin Hou

**Affiliations:** 1 State Key Laboratory for Biology of Plant Diseases and Insect Pests, Institute of Plant Protection, Chinese Academy of Agricultural Sciences, Beijing, 100193 China; 2 College of Plant Protection, Hunan Agricultural University, Changsha, 410128 China; 3 Southern Regional Collaborative Innovation Center for Grain and Oil Crops in China, Changsha, 410128 China

## Abstract

The brown planthopper (BPH), *Nilaparvata lugens* (Stål), is a migratory and destructive sucking insect pest of rice. Silicon (Si) amendment to plants can confer enhanced resistance to herbivores and is emerging as a novel approach for pest management. In the present study, we tested the effects of Si addition at 0.16 (low) and 0.32 (high) g Si/kg soil on sucking behaviors and population growth in BPH. Si amendment increased Si content in rice stems and extended non-probing event and phloem puncture followed by sustained phloem ingestion over that in the no-Si-addition control. High Si addition rate prolonged the stylet pathway and the time needed to reach the first phloem puncture, shortened durations of phloem puncture and phloem ingestion, and decreased the proportion of individuals that produced sustained phloem ingestion. BPH female feeding on and preference for plants with the high Si addition rate were also reduced. As a result, Si application significantly decreased BPH population growth rates while increased population doubling time. These results indicate that Si amendment, especially at the high rate, confers enhanced rice plant resistance to BPH through impairment of BPH feeding. Our results highlight the potential of Si amendment as an alternative for BPH management.

## Introduction

Rice (*Oryza sativa* L.), accounting for almost one-fifth of the total world cropland under cereal, is attacked by about 800 species of insect pests in both field and storage^1^. The brown planthopper (BPH), *Nilaparvata lugens* (Stål) (Hemiptera: Delphacidae), is one of the migratory and most destructive pests of rice and a substantial threat to rice production^2^. The BPH is a monophagous herbivore, and is a typical vascular feeder. It sucks the phloem sap from leaf sheath of rice plants using its stylet, and causes direct damage to rice plants. BPH can also cause indirect damage to rice plants through the transmission of plant viruses^2^. Extensive use of chemical insecticides has been the common practice for control of BPH, which has resulted in many problems, including toxicity to natural enemies^3^, increased production cost, and possible long term agro-ecosystem and human health damage^4^. Breeding and utilization of resistant rice varieties is one of the most economical and effective strategies in controlling this insect pest. However, up to now only a few BPH-resistant rice varieties have been developed and cultivated in the rice cultivation areas^5^. Thus, there is an urgent need to develop effective and ecologically sound alternative methods to improve the pest control. Silicon (Si) amendment may be one of such potential alternatives^6^.

Si is generally not considered an essential element for plant growth, but due to its important role in plant nutrition, particularly under stressful conditions, it is now recognized as a “beneficial substance” or “quasi-essential”^7^. Enhanced resistance to both sucking and chewing insect pests following artificial Si application has been observed in a wide variety of plant species^8^.

Si-mediated resistance may be realized through priming chemical defense reaction in plants^9–11^, intensified physiological and mechanical barriers resulting from amorphous silica deposition in plant tissues^12–14^, or reduced digestion efficiency in herbivores^12, 15^. Additionally, Si amendment can have an influence on indirect plant defense through augmented release of herbivore-induced plant volatiles that attract natural enemies of the attacking pests^16^.

For the sucking herbivorous BPH, rice lines treated hydroponically with high silicon concentrations reduced performance of BPH^17^. However, the influence of Si amendment on BPH sucking behaviors and population growth has to be further determined.

The sucking behaviors of sucking insects comprise of a sequence of several steps where physical and chemical factors of plant tissues may have an influence^18^. Observation of stylet penetration (probing) in the plant tissues can help locate resistance factors or mechanisms^19^. Electrical penetration graph (EPG) has emerged to be a powerful technique for such observation^19^. With EPG waveforms correlated with particular stylet activities in BPH^18^, it was observed that BPH spent more time wandering over rice plants carrying resistance genes but less time ingesting phloem than they do on susceptible plants, and phloem ingestion was frequently interrupted on resistant plants^20, 21^. In wheat plants amended with Si, both the feeding time of *Schizaphis graminum* and the percentage of insects that fed on phloem sap were reduced^22, 23^, which may result from the fact that the stylets of *S. graminum* were withdrawn more often on wheat plants treated with Si^24^.

The objective of this study was to assess the effects of Si addition to a susceptible rice variety on BPH sucking behaviors and population parameters. Such information can provide evidence of Si-mediated resistance to BPH and furthers our understanding of the ways for the Si-mediated plant resistance.

## Results

### Si content in rice stem

Silicon application at transplanting significantly increased Si content in stems of the plants 40 days after transplanting (DAT) by 42.9% (Tukey HSD test, *P* < 0.001) and 19.1% (Tukey HSD test, *P* = 0.013) at the high and low Si addition rates over that (14 mg/g DW) in the control, respectively.

### Sucking behavior

BPH sucking behaviors recorded for 6 h by EPG were summarized in Table 1. The total duration of non-probing (np) event on the plants with high Si addition was much longer than that in the control (Tukey HSD test, *P* = 0.029). The mean duration of np was also longer when the insects were feeding on plants amended with Si than in the control (*F* = 7.693, df = 2, 38, *P* = 0.002). The pathway event, which comprises of penetration initiation (N1), salivation and stylet movement (N2), and extracellular stylet activity near phloem region (N3), was significantly extended on the plants with the high Si addition rate (156.5 min) over that in the control (118.6 min) (Tukey HSD test, *P* = 0.004). Phloem puncture (N4a) event lasted for a shorter duration on the plants with the high Si addition rate (57.5 min) than in the control (81.4 min) (Tukey HSD test, *P* = 0.024). Phloem sap ingestion (N4b) was shorter in both total and mean durations on the plants with the high Si addition rate than in the control (Tukey HSD test, *P* ≤ 0.011).

**Table 1:** Feeding behaviors of *Nilaparvata lugens* recorded by EPG on rice plants amended with Si or not over a 6-h recording period.

Si treatment (g Si/kg soil)	Non-probing (np)	Pathway	Phloem puncture (N4a)	Phloem ingestion (N4b)
**Total duration (min) of each EPG waveform**				
0	6.1 ± 1.9 a	118.6 ± 3.2 a	81.4 ± 4.3 a	54.3 ± 6.9 a
0.16	12.1 ± 3.3 ab	144.9 ± 8.6 ab	65.8 ± 6.5 ab	42.8 ± 7.0 ab
0.32	21.0 ± 5.2 b	156.5 ± 9.2 b	57.5 ± 6.8 b	26.2 ± 5.5 b
ANOVA statistics	*F* = 3.70, df = 2,38, *P* = 0.035	*F* = 6.10, df = 2,38, *P* = 0.005	*F* = 3.89, df = 2,38, *P* = 0.029	*F* = 4.86, df = 2,38, *P* = 0.014
**Mean duration (min) of each EPG waveform**				
0	1.3 ± 0.2 a	19.3 ± 1.8 ab	23.1 ± 2.4 a	45.0 ± 5.1 a
0.16	2.2 ± 0.3 b	17.3 ± 1.6 a	12.7 ± 1.8 b	33.4 ± 3.9 ab
0.32	2.8 ± 0.3 b	28.5 ± 3.8 b	20.3 ± 2.7 ab	22.6 ± 3.4 b
ANOVA statistics	*F* = 7.69, df = 2,38, *P* = 0.002	*F* = 4.94, df = 2,38, *P* = 0.013	*F* = 4.99, df = 2,38, *P* = 0.012	*F* = 7.29, df = 2,38, *P* = 0.002

Data are expressed as mean ± SE. Data in a column followed by different letters are significantly different (Tukey’s multiple range test, *P* = 0.05). The data are averages of 12–14 recordings per treatment.

**Figure 1:**
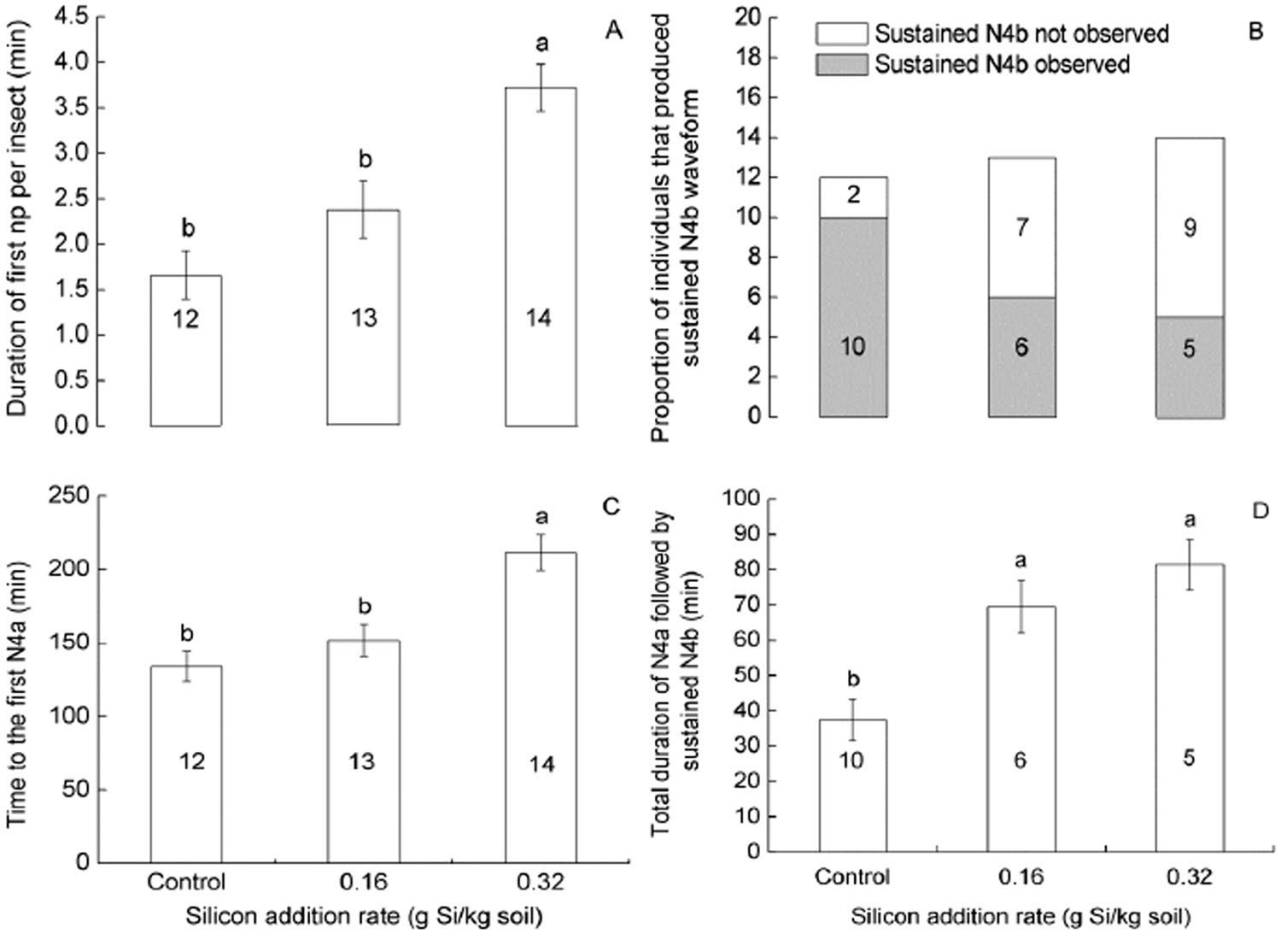
Feeding behaviors of *Nilaparvata lugens* recorded by EPG on rice plants amended with Si or not over a 6-h recording period. (**A**) Duration of first np per insect, (**B**) proportion of individuals that produced sustained N4b (>10 min), (**C**) time to the first N4a, (**D**) total duration of N4a followed by sustained N4b. In panels A, C and D, values (means ± SE) labelled with different letters are significantly different (Tukey’s multiple range test, *P* = 0.05), and the figures within the bars indicate numbers of replication. In panel B, the figures within the bars indicate numbers of insects in which sustained N4b was observed or not.

High Si addition rate significantly extended the duration of the first np (*F* = 14.272, df = 2, 38, *P* < 0.001; Fig. 1A) and the time needed to reach the first N4a (*F* = 13.25, df = 2, 38, *P* < 0.001; Fig. 1C) as compared with low Si addition rate and the control. The proportion of individuals that produced sustained N4b (longer than 10 min) waveform was significantly lower in the high Si addition rate than in the control (Chi-Square test, *P* = 0.014; Fig. 1B). Total duration of N4a followed by sustained N4b was longer on the plants with Si amendment as compared with the control (*F* = 12.168, df = 2, 20, *P* < 0.001; Fig. 1D).

### Honeydew excretion and host acceptance

**Figure 2:**
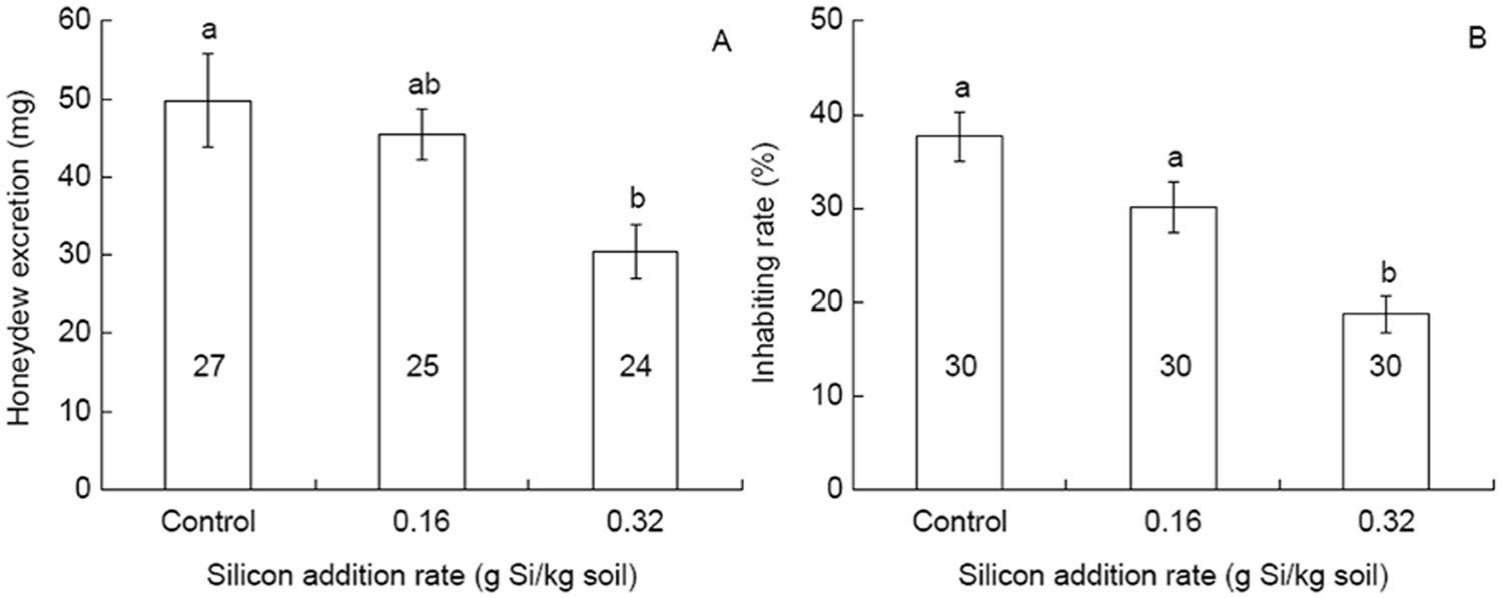
Effects of silicon addition to rice plants (var. TN1) on honeydew excretion (**A**) and host acceptance (**B**) in *Nilaparvata lugens* adults. Values are expressed as means ± SE. Bars with different letters are significantly different (Tukey’s multiple range test, *P* = 0.05). Figures within the bars indicate numbers of replication.

BPH honeydew excretion measured in 72 h was less (by 39.0%) in the high Si addition treatment than in the control (*F* = 4.929, df = 2, 75, *P* = 0.01; Fig. 2A). Si addition showed a deterrence effect on BPH host acceptance (*F* = 14.623, df = 2, 89, *P* < 0.001; Fig. 2B), adults accepted the rice plants amended with the high rate of Si less (by 19.0%) than they did for the control plants.

### Population parameters

Using life table analysis, population parameters of BPH feeding on rice plants amended with Si or not were established (Table 2). Si treatment significantly reduced the intrinsic rate of increase (*F* = 43.621, df = 2, 8, *P* < 0.001), the finite rate of increase (*F* = 43.943, df = 2, 8, *P* < 0.001), and the net reproduction rate (*F* = 344.251, df = 2, 8, *P* < 0.001) in comparison with the control. The mean generation time ranging from 34 to 36 d was significantly reduced in the high Si treatment compared to the control (Tukey HSD test, *P* = 0.027). The population doubling time was longer in Si amended treatments than in the control (*F* = 36.767, df = 2, 8, *P* < 0.001).

**Table 2:** Population parameters of *Nilaparvata lugens* feeding on rice plants amended with Si or not.

Si treatment (g Si/kg soil)	*r*_*m*_	*λ*	*R*_*0*_	*T*	*DT*
0	0.10 ± 0.0007 a	1.10 ± 0.0008 a	35.8 ± 0.43 a	36.2 ± 0.28 a	7.0 ± 0.05 a
0.16	0.09 ± 0.0003 b	1.09 ± 0.0003 b	21.2 ± 0.64 b	35.7 ± 0.28 ab	8.1 ± 0.03 b
0.32	0.09 ± 0.0018 b	1.09 ± 0.0020 b	19.3 ± 0.34 b	34.2 ± 0.55 b	8.0 ± 0.16 b

Data are expressed as mean ± SE. Data in a column followed by different letters are significantly different (Tukey’s multiple range test, *P* = 0.05). The observation was made to three populations in each treatment. *r*_*m*_, intrinsic rate of increase (eggs per female per d); *λ*, finite rate of increase (population growth rate per d); *R*_*0*_, net reproductive rate (eggs per female); *T*, mean generation time (d); *DT*, population doubling time (d).

## Discussion

Enhanced plant defense associated with Si amendment has been previously reported in insect herbivores^15, 17, 23–26^. In the present study, we observed that silicon application to rice plants reduced population growth rates and extended population doubling time in BPH, and thus conferred resistance. Similar effects of Si amendment on herbivore populations were also reported in aphids *Myzus persicae* ^27^ and *Sitobion avenae* (F.) (Hemiptera: Aphididae)^28^. It can be reasoned that the poor population performance of BPH on plants amended with Si results from reduced individual performance, such as decrease in adult fecundity and nymphal survival rate (Fig. S1), as reported for *N. Lugens* ^17^ and *S. avenae* ^28^.

Silicon may enhance plant resistance to herbivores in several ways. In the current study, we show that Si addition impaired phloem sap feeding by BPH. We observed a reduced time in ingesting phloem (N4b) by BPH on silicon-treated plants (Table 1) and reduced proportion of individuals that produced sustained (>10 min) phloem ingestion (Fig. 1B). These results parallel to those results observed in resistant rice varieties^20, 21^, where BPH spent less time ingesting phloem on plants carrying the resistance genes *Bph14* and *Bph15* than they did on susceptible plants. Our results also correspond to those by Pereira *et al*.^22^ and Costa *et al*.^23^, who found that the greenbug *S. graminum* showed a reduction in the total duration of phloem phase, and a decrease in sap ingestion time on silicon-treated wheat plants. Shortened duration of the phloem phase (both N4a and N4b, Table 1) under Si treatment suggests that the insect’s effort in ingestion of phloem sap is repressed. This might explain the decreased BPH feeding (honeydew excretion) on silicon treated plants. However, total duration of N4a followed by sustained N4b is longer when Si was added than when Si was not added (Fig. 1D). This indicates that, for a sustained phloem ingestion (N4b) to occur on plants amended with Si, the insects have to spend longer time in phloem puncture (N4a) than on plants not amended with Si. The Si-mediated reduced BPH feeding may be associated with callose deposition. Callose is a plant polysaccharide, produced in response to herbivorous feeding and deposited on the sieve plates^20^. Callose deposition hampers phloem transportation and prevents phloem sap ingestion by sucking insects^20^. Silicon amendment might have primed callose synthesis and deposition and thus hampered phloem sap ingestion by BPH. Silicon-induced resistance in wheat against the powdery mildew *Blumeria graminis* f. sp. *tritici* is associated with increased accumulation of callose^29^. The metabolism and deposition of callose in response to BPH feeding and Si amendment have to be further investigated in rice. And, soluble silicic acid extracted from rice leaf sheaths reduced BPH feeding^30, 31^ and it can be argued that concentration of silicic acid, the chemical form of silicon available to biological systems^31^ in both soil and sheath phloem sap, has increased in response to Si amendment and plays a role in sucking inhibition. A series of biochemical and physiological changes in plants resulted from Si amendment may interfere with herbivore feeding. Si can mobilize the accumulation of phenolic compounds, chitinases and peroxidases^32, 33^, act as a candidate for association with cell wall components (polysaccharides and cell wall–associated proteins)^34^, and is involved in Si-aromatic ring associations between lignin and carbohydrate in rice^35^. The role of these biochemical and physiological changes in inhibiting BPH feeding has to be further tested. Still, increased hardness and toughness of plant tissues (epidermis and mesophyll) as a result of increased silica deposition^8^ may discourage feeding activities and also contribute to the Si-mediated reduced BPH feeding.

In addition to feeding inhibition, we also recorded antixenosis effect of Si-amended rice plants on BPH, as indicated by deterrence of BPH settlement on plants with the high Si addition rate (Fig. 2B). Similar results were reported previously for white-backed planthopper^36^ and BPH^17^. Increased silica deposition, in the form of phytoliths in hairs, trichomes and spines, resulted from Si amendment^8^, might have deterred BPH settlement on Si-amended plants. Similar role of high density and large volume of silica is reported in rice cultivars resistant to the small brown planthopper *Laodelphax striatellus* Fallén^37^.

Further, Si amendment resulted in longer time spent in non-probing and stylet pathway activities (Table 1), as in the greenbug *S. graminum* ^22–24^. The increased time in non-probing and stylet pathway activities associated with Si addition may be a result of increased hardness and toughness of plant tissues (epidermis and mesophyll) because of silica deposition in plant tissues^8^. Enhanced soluble silicic acid in rice leaf sheaths and the above mentioned biochemical and physiological changes as a result of Si amendment may change the phloem sap and plant tissue property and also contribute to the longer time spent in stylet pathway activities, although it deserves further test.

In conclusion, it is obvious that Si addition enhances rice plant resistance to BPH through impairment of phloem sap feeding, deterrence of settlement on plants, and elongation of non-probing and stylet pathway activities. This study indicates that use of silicon-based fertilizers holds a potential in BPH management.

## Methods

### Plants and Si Treatments

The plants and Si treatments were largely the same as described by Han *et al*.^15^. Briefly, germinated seeds of a susceptible variety (Taichung Native 1, TN1) were sown in soil without addition of calcium silicate. Rice seedlings were transplanted to 10 L PVC pots 25 days after sowing at two 2-seedling hills per pot in a glasshouse at Guilin Experiment Station for Crop Pests (25°36′00″ N, 110°41′24″ E), Ministry of Agriculture, China. The pots each were filled with 4.2 kg dry soil, amended with calcium silicate (soluble Si ≥ 11.7%, Shanxi Fubon Siliconfat Co., Ltd, Jinzhong, China) at a low rate of 0.16 or a high rate of 0.32 g Si/kg soil or left untreated (control). Soil used for the plants were obtained from the fields of the station, whose chemical properties were described previously^15^. All the pots were treated with urea (N ≥ 46.4%), diammonium phosphate (N = 16.0%; P_2_O_5_ = 44.0%) and potassium chloride (K_2_O ≥ 60.0%) at a rate of 0.37 g kg^−1^ soil, 0.25 g kg^−1^ soil and 0.35 g kg^−1^ soil, respectively. Urea was applied into soil 3 days before transplanting or top dressing at tillering, heading and milk stages at a ratio of 4:3:2:1, diammonium phosphate and calcium silicate were all incorporated into the soil 3 days before transplanting, and potassium chloride was supplied in soil 3 days before transplanting or top dressing at heading stage at a ratio of 2:1. The pots were arranged randomly in the glasshouse. Watering was administered as necessary and water level in the pots was always below the upper edge. Pesticides were not used throughout the experiment. Rice plants at 40 days after transplanting (DAT) were used in the experiments. Si content in stems (including culm and leaf sheath, about 200 g) from 40 DAT rice plants not fed by BPH was measured by the colorimetric molybdenum blue method^38^.

### Insects

BPH adults were collected in late July, 2014 from paddy fields at the experiment station. BPH were reared on the control plants without exposure to any insecticide within 80-mesh insect proof cages (60 by 60 by 60 cm) in the glasshouse at the experiment station. Newly hatched nymphs (<24 h) and newly emerged adult females (<24 h) were used in the experiments.

### EPG recording and parameters of sucking behavior

BPH feeding behavior was recorded using a GiGA-8 DC electrical penetration graph (EPG) amplifier system (Wageningen Agricultural University, Wageningen, The Netherlands) as reported by Lei *et al*.^39^. Briefly, a newly emerged macropterous BPH female (<24 h) starved for 1 h was attached to one end of a gold wire (18 μm in diameter and 3–5 μm in length) at the dorsal thorax of the insect using a drop of water-soluble silver glue. The wired insect was then connected to the amplifier through a copper nail that was inserted into the EPG probe and placed on a rice leaf sheath of a 40-DAT plant. A copper wire (2 mm diameter × 10 cm length) connected to the amplifier and vertically inserted into the pot soil was used as the plant electrode. Each insect was continuously recorded for 6 h. A different BPH and plant was used for each recording until 12–14 recordings were reached for each treatment. The EPG recording was conducted in a quiet room with ambient conditions of 27 ± 2 °C and RH 70 ± 5%. The recorded signals were analysed using PROBE 3.4 software (Wageningen University, Wageningen, The Netherlands).

The EPG waveforms were classified according to Seo *et al*.^18^ and Lei *et al*.^39^ into: non-probing (np), penetration initiation (N1), salivation and stylet movement (N2), extracellular activity near the phloem region (N3), intracellular activity in the phloem tissue (N4a), phloem sap ingestion (N4b), and stylets in the xylem tissue (N5), with N1, N2 and N3 pooled together as pathway event. A probe was defined to start from the insect stylet insertion to the stylet withdrawal. Six parameters were obtained from the EPG recordings^40, 41^: waveform duration per insect (min), which is the sum of the durations of each event of all the waveforms made by an insect; waveform duration per event (min), which is the sum of the durations of each event for a particular waveform divided by the number of events of that particular waveform; duration of first np per insect (min); time to the first N4a (min); proportion of individuals that produced the sustained N4b (longer than 10 min) waveform; and total duration of N4a followed by sustained N4b (min). All the parameters were averaged across the number of insects or events (in the sense of Backus *et al*.^41^).

### Honeydew excretion

Honeydew excretion that indicates feeding amount was measured using a parafilm sachet method^42^. A newly emerged macropterous female starved for 2 h was placed in a parafilm sachet (3.5 by 3.5 cm) fixed onto the stem of a 40-DAT plant. The insect was allowed to feed for 72 h and then removed from the sachet. Then the sachet was weighed immediately, and after removal of honeydew, weighed again. This allowed the net weight of honeydew excretion to be obtained. The experiment was performed in a laboratory under natural conditions. Each insect served as a replicate, 30 insects were tested for each treatment.

### Host acceptance

The influence of Si addition on BPH host acceptance was tested using caged 40-DAT rice plants. The potted plants were incubated in insect-proof cages and any arthropods on them were removed before their use in the tests. The tillers of each potted plant were thinned to leave only four. Two pots were randomly selected from each of the three treatments, totaling six pots. The potted plants were arranged alternatively according to treatment in a circle (diameter 100 cm) within a cylindrical cage (diameter 120 cm, height 100 cm) made from iron wire and 80-mesh nylon netting. The pots were separated at about 60 cm from each other. Thirty three-day old macropterous BPH females were released from a Petri dish (9 cm in diameter and 2 cm in height) placed in the center of the cage. The insects settling on each plant were counted at 72 h after release. The tests were performed in a glasshouse, under natural light and temperature conditions and repeated 30 times. Between repetitions, the plants were rotated to balance any effects from unforeseen asymmetries.

### Population parameters

To obtain life table parameters of population of BPH feeding on rice plants amended with Si or not, 10 first instars (<24 h) were transferred into a glass tube (5 cm in diameter, 40 cm in height), where there were two 40-DAT plants transferred from the potted rice hills together with the soil amended with Si or not. The rice plants therein were replaced every 5 d with plants of the same treatment. A slice of cut-out sponge was lined on the bottom of the glass tube and circled the rice stem to secure the rice plant and to prevent drowning of the planthopper. The top of the glass tube was also sealed with sponge to prevent insect escape. The insects were left to develop in a climate chamber (RXZ-160A, Ningbo Dongnan Instruments Co., Ltd, Ningbo, China) at 27 ± 1 °C, 70 ± 5% relative humidity (RH) and a photoperiod of 16:8 (L:D) h. The glass tubes were observed daily to record developmental stage and numbers of surviving nymphs until adult emergence. The emerged adults were sorted daily according to wing form and sex. The observation of nymphal survival and sex ratio was repeated for 3 times, with each 10 insect cohort as a replicate. Newly emerged macropterous adults (<24 h) of the same treatment were paired and their longevity and fecundity were investigated using the same arena as for the nymphs. The observation was replicated 30 times for each treatment, with one pair of adults as a replicate. Life table parameters, including the intrinsic rate of increase (*r*_*m*_), finite rate of increase (*λ*), net reproductive rate (*R*_*0*_), and mean generation time (*T*) were calculated for BPH populations of different Si treatments using the computer program^43^.

### Statistics analysis

All data were subjected to one-way analysis of variance (ANOVA), followed by Tukey’s multiple range test (*P* = 0.05) for significant differences between treatments^40^. Percentage data were arcsine square-root transformed, and homogeneity of variance of all data was tested before ANOVA^44^. Life table parameters were also subjected to ANOVA for treatment effects and means were separated using Tukey’s multiple range tests. Proportion of individuals that produced the sustained N4b waveform was compared among the treatments using a Chi-square 2 × 2 goodness of fit test or a Fisher’s Exact Test when the expected values were lower than 5.

## Electronic supplementary material

Supplementary info file
