# Supplementary material for: Silicon amendment to rice plants impairs sucking behaviors and population growth in the phloem feeder Nilaparvata lugens (Hemiptera: Delphacidae)
Source: Sci Rep. 2017 Apr 24;7:1101. doi: 10.1038/s41598-017-01060-4 (PMC5430648; doi:10.1038/s41598-017-01060-4)
Supplement: Supplementary file 1 — Supplementary info file [file 41598_2017_1060_MOESM1_ESM.pdf]

Silicon amendment to rice plants impairs sucking behaviors and population growth in a phloem feeder *Nilaparvata lugens* (Hemiptera: Delphacidae)

Lang Yang[1,3], Yongqiang Han[1,2,3], Pei Li[1,3], Lizhang Wen[2], and Maolin Hou[1,3,*]

[1] State Key Laboratory for Biology of Plant Diseases and Insect Pests, Institute of Plant Protection, Chinese Academy of Agricultural Sciences, Beijing 100193, China

[2] College of Plant Protection, Hunan Agricultural University, Changsha 410128, China

[3]Southern Regional Collaborative Innovation Center for Grain and Oil Crops in China, Changsha 410128, China

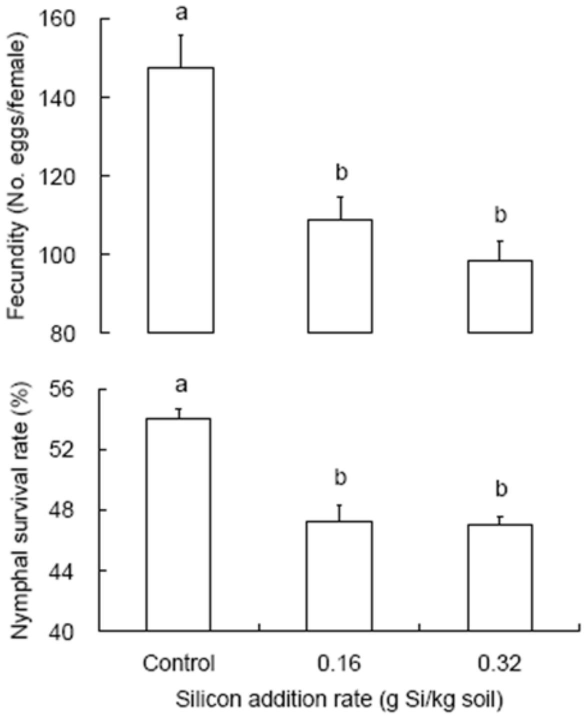

Figure S1. Fecundity (A) and nymphal survival rate (B) of *Nilaparvata lugens* feeding on rice plants amended with Si or not. Values are expressed as means ± SE. Bars with different letters are significantly different (Tukey's multiple range test, $P = 0.05$). Observation of fecundity was repeated for 30 times and of nymphal survival rate, for 3 times.

Supplementary dataset

Table S1 Silicon content in 40 DAT rice plants

treatment	SiO_2 (mg/g DW)	Si (mg/g DW)
0	31.58	14.74
0	26.81	12.51
0	31.58	14.74
1	36.36	16.97
1	36.36	16.97
1	34.52	16.11
2	40.77	19.03
2	43.35	20.23
2	44.45	20.74
0 = control, 1 = 0.16 g Si/kg soi, 2 = 0.32 g Si/kg soil		

Table S2 BPH adult settlement on rice plants in a choice test

treatment	% settlement	treatment	% settlement	treatment	% settlement
0	23.33	1	30.00	2	26.67
0	23.33	1	13.33	2	23.33
0	26.67	1	16.67	2	13.33
0	36.67	1	13.33	2	3.33
0	16.67	1	13.33	2	10.00
0	16.67	1	6.67	2	10.00
0	40.00	1	0.00	2	10.00
0	16.67	1	23.33	2	20.00
0	33.33	1	23.33	2	26.67
0	30.00	1	30.00	2	26.67
0	36.67	1	43.33	2	36.67
0	50.00	1	36.67	2	33.33
0	36.67	1	20.00	2	13.33
0	40.00	1	30.00	2	16.67
0	26.67	1	36.67	2	16.67
0	46.67	1	23.33	2	10.00
0	33.33	1	36.67	2	16.67
0	43.33	1	43.33	2	20.00
0	73.33	1	73.33	2	3.33
0	60.00	1	36.67	2	6.67
0	46.67	1	33.33	2	46.67
0	76.67	1	56.67	2	6.67
0	40.00	1	26.67	2	16.67
0	36.67	1	23.33	2	36.67
0	40.00	1	40.00	2	6.67
0	43.33	1	50.00	2	13.33
0	30.00	1	33.33	2	20.00
0	23.33	1	20.00	2	26.67
0	46.67	1	33.33	2	23.33
0	36.67	1	36.67	2	20.00
0 = control, 1 = 0.16 g Si/kg soi, 2 = 0.32 g Si/kg soil					

Table S3 Honeydew excretion of BPH feeding on rice plants

treatment	honeydew excretion (mg)	treatment	honeydew excretion (mg)	treatment	honeydew excretion (mg)
0	10.1	1	12.4	2	5.9
0	15.8	1	15.4	2	6.1
0	18.2	1	18.8	2	7.0
0	18.4	1	25.5	2	9.9
0	20.0	1	32.7	2	12.6
0	26.9	1	33.9	2	14.4
0	32.6	1	35.7	2	16.5
0	34.4	1	38.7	2	17.7
0	34.6	1	39.3	2	19.3
0	35.7	1	40.1	2	25.2
0	36.3	1	41.7	2	31.2
0	37.0	1	43.6	2	32.2
0	39.4	1	49.4	2	33.5
0	41.0	1	49.5	2	35.4
0	43.6	1	51.2	2	35.5
0	44.6	1	52.9	2	38.2
0	45.3	1	53.9	2	38.4
0	45.5	1	53.9	2	39.9
0	48.7	1	55.4	2	41.4
0	48.7	1	58.9	2	41.5
0	65.4	1	60.4	2	47.8
0	79.6	1	61.0	2	55.6
0	90.7	1	69.6	2	56.5
0	92.8	1	70.7	2	67.1
0	99.5	1	71.4		
0	105.3				
0	134.8				
0 = control, 1 = 0.16 g Si/kg soi, 2 = 0.32 g Si/kg soil					

Table S4 Mean duration of behavioral events recorded by EPG for BPH feeding on rice plants

treatment	mean duration of np (min)	mean duration of Nc (N1 + N2 + N3）(min)	mean duration of N4a (min)	mean duration of N4b (min)
0	0.37	12.54	23.07	50.11
0	0.8	13.73	13.33	39.79
0	0.84	16.04	15.09	59.63
0	1.09	12.40	26.46	30.29
0	1.1	13.00	11.13	21.35
0	1.32	20.32	18.79	34.91
0	1.33	18.05	18.94	28.31
0	1.33	19.50	21.59	30.90
0	1.43	23.46	26.38	41.91
0	1.56	28.73	28.06	76.60
0	2.07	29.79	35.03	71.56
0	2.54	24.22	38.87	54.16
1	0.83	13.49	0.00	14.15
1	0.87	17.70	11.11	49.81
1	1.51	8.61	11.19	25.48
1	1.65	11.24	12.93	21.37
1	1.7	16.73	5.16	24.39
1	1.74	12.62	15.79	59.68
1	2.19	13.61	8.38	25.10
1	2.28	16.70	8.60	23.50
1	2.35	17.93	13.04	24.29
1	2.35	19.46	16.92	54.08
1	3.12	21.81	20.86	31.88
1	3.56	29.74	23.15	44.49
1	4.89	24.62	17.74	35.60
2	2.54	37.41	0.00	31.10
2	2.85	28.85	11.48	22.56
2	2.86	39.76	24.03	13.77
2	2.92	11.28	34.04	23.10
2	2.95	12.53	16.03	9.90
2	4.28	13.27	10.40	8.76
2	4.33	15.62	15.07	17.13
2	1.41	17.58	15.70	14.01
2	4.47	22.59	16.48	9.73
2	1.57	25.57	21.10	25.29
2	1.96	26.50	34.40	26.65
2	2.43	43.91	35.33	54.00
2	1.4	56.84	29.15	41.31
2	2.73	46.62	20.48	18.98

0 = control, 1 = 0.16 g Si/kg soi, 2 = 0.32 g Si/kg soil

Table S5 Total duration of behavioral events recorded by EPG for BPH feeding on rice plants

treatment	total duration of np (min)	total duration of Nc (N1 + N2 + N3）(min)	total duration of N4a (min)	total duration of N4b (min)
0	0.37	97.49	71.33	22.98
0	0.84	107.08	67.29	42.99
0	1.43	121.95	70.39	35.11
0	1.56	115.04	62.89	58.25
0	1.61	123.38	61.03	51.13
0	3.96	135.32	88.87	24.03
0	4.34	126.07	93.97	41.59
0	5.08	108.30	100.13	41.71
0	6.62	112.33	105.51	75.60
0	10.63	118.17	80.10	83.87
0	16.59	126.09	90.10	90.56
0	19.96	132.04	85.37	84.16
1	0.83	98.71	52.23	43.85
1	1.51	110.49	62.21	44.38
1	3.56	137.75	42.37	19.90
1	4.33	120.89	47.37	21.44
1	4.89	108.25	26.40	18.58
1	6.25	124.33	46.43	48.10
1	6.79	144.92	65.19	22.77
1	8.75	166.53	94.63	13.50
1	13.67	176.04	99.44	28.59
1	16.42	179.84	100.55	51.88
1	23.16	196.70	78.30	83.49
1	24.40	168.20	80.22	77.60
1	42.34	151.32	60.28	82.25
2	0.86	102.84	70.21	15.02
2	0.92	126.45	54.43	14.75
2	2.69	152.16	20.80	24.28
2	2.85	148.74	24.18	31.04
2	3.93	121.55	30.17	9.84
2	7.65	125.89	42.20	4.52
2	9.42	126.61	45.20	14.16
2	21.33	145.30	48.48	21.10
2	32.69	172.31	65.90	0.00
2	32.83	180.88	68.79	26.65
2	34.56	215.75	78.31	49.30
2	34.72	182.00	56.00	75.86
2	52.51	206.42	109.88	51.31
2	57.00	184.08	90.95	28.98
0 = control, 1 = 0.16 g Si/kg soi, 2 = 0.32 g Si/kg soil				

Table S6 Duration of the first non-probing event recorded by EPG for BPH feeding on rice plants

treatment	duration of the first np (min)	treatment	duration of the first np (min)	treatment	duration of the first np (min)
0	2.96	1	2.96	2	2.96
0	1.56	1	2.15	2	2.96
0	1.23	1	4.89	2	3.64
0	1.78	1	3.56	2	2.15
0	2.58	1	3.17	2	4.45
0	1.49	1	2.12	2	2.3
0	3.57	1	2.38	2	4.13
0	0.84	1	1.29	2	3.92
0	0.71	1	1.29	2	3.89
0	0.37	1	2.75	2	4.77
0	1.43	1	2.1	2	4.86
0	1.39	1	1.51	2	4.33
		1	0.83	2	2.7
				2	5.06
0 = control, 1 = 0.16 g Si/kg soi, 2 = 0.32 g Si/kg soil					

Table S7 Time needed to reach the first N4a event recorded by EPG for BPH feeding on rice plants

treatment	time needed to the first N4a (min)	treatment	time needed to the first N4a (min)	treatment	time needed to the first N4a (min)
0	64.34	1	103.94	2	214.42
0	107.88	1	158.77	2	276.17
0	108.21	1	189.62	2	229.99
0	120.43	1	101.33	2	172.71
0	145.55	1	161.61	2	275.15
0	163.35	1	209.75	2	149.36
0	146.25	1	84.03	2	162.84
0	125.06	1	131.34	2	164.21
0	113.35	1	174.00	2	218.51
0	155.13	1	200.54	2	259.01
0	201.13	1	128.60	2	173.09
0	158.93	1	160.00	2	200.00
		1	170.76	2	275.93
				2	189.90
0 = control, 1 = 0.16 g Si/kg soi, 2 = 0.32 g Si/kg soil					

Table S8 Total duration of N4a followed by N4b recorded by EPG for BPH feeding on rice plants

treatment	total duration of N4a followed by N4b (min)
0	33.33
0	17.41
0	19.36
0	22.60
0	23.20
0	37.89
0	40.12
0	60.20
0	50.70
0	82.73
1	67.45
1	48.19
1	73.18
1	81.41
1	96.27
1	67.01
2	68.27
2	67.32
2	77.48
2	89.61
2	104.82
treatment: 0 = control, 1 = 0.16 g Si/kg soi, 2 = 0.32 g Si/kg soil	

Table S9 Total duration of N4a followed by sustained N4b recorded by EPG for BPH feeding on rice plants

treatment	total duration of N4a followed by sustained N4b (min)
0	17.41
0	19.53
0	22.60
0	23.33
0	30.01
0	37.89
0	40.11
0	52.73
0	60.20
0	70.54
1	50.68
1	48.19
1	73.18
1	81.41
1	96.27
1	67.01
2	68.27
2	67.32
2	77.48
2	89.61
2	104.82
treatment: 0 = control, 1 = 0.16 g Si/kg soi, 2 = 0.32 g Si/kg soil	

Table S10 Frequency of sustained N4b recorded by EPG for BPH feeding on rice plants

treatment	occurrence of sustained N4b	frequency of sustained N4b
0	0	2
0	1	10
1	0	7
1	1	6
2	0	9
2	1	5
treatment: 0 = control, 1 = 0.16 g Si/kg soi, 2 = 0.32 g Si/kg soil		
occurrence: 0 = no, 1 = yes		

Table S11 Frequency of sustained N4b recorded by EPG for BPH feeding on rice plants

treatment	probing duration per insect (min)	treatment	probing duration per insect (min)	treatment	probing duration per insect (min)
0	340.04	1	330.64	2	207.49
0	343.41	1	335.60	2	303.00
0	349.37	1	336.84	2	345.28
0	353.38	1	343.58	2	345.44
0	354.92	1	346.33	2	347.17
0	355.66	1	351.25	2	347.31
0	356.04	1	353.21	2	350.58
0	358.39	1	353.75	2	352.35
0	358.44	1	355.11	2	356.07
0	358.57	1	355.67	2	357.15
0	359.16	1	356.44	2	357.31
0	359.63	1	358.49	2	358.67
		1	359.17	2	359.08
				2	359.14
0 = control, 1 = 0.16 g Si/kg soi, 2 = 0.32 g Si/kg soil					

Table S12 Life table parameters of BPH feeding on rice plants

	survival rate						
treatment	egg	1st instar	2nd instar	3rd instar	4th instar	5th instar	female rate
0	0.874	0.900	0.889	0.929	0.885	0.804	0.51
0	0.874	0.914	0.906	0.931	0.889	0.792	0.52
0	0.874	0.930	0.894	0.932	0.873	0.813	0.51
1	0.842	0.890	0.877	0.912	0.865	0.733	0.48
1	0.842	0.904	0.894	0.915	0.870	0.745	0.49
1	0.842	0.892	0.879	0.914	0.887	0.766	0.50
2	0.858	0.917	0.879	0.914	0.868	0.739	0.50
2	0.858	0.903	0.892	0.897	0.865	0.733	0.49
2	0.858	0.890	0.908	0.915	0.870	0.745	0.47
treatment	eggs/ female	population trend index	generation time (T, d)	intrinsic rate of increase (r_m)	population doubling time (DT, d)	finite rate of increase（λ）	net reproductive rate (R_0)
0	147.6	34.99	36.04	0.099	7.027	1.104	34.990
0	147.6	36.10	35.78	0.100	6.915	1.105	36.103
0	147.6	36.41	36.73	0.098	7.083	1.103	36.410
1	108.8	20.08	35.31	0.085	8.158	1.089	20.082
1	108.8	21.34	35.61	0.086	8.065	1.090	21.340
1	108.8	22.29	36.24	0.086	8.092	1.089	22.290
2	98.7	19.98	33.25	0.090	7.695	1.094	19.983
2	98.7	18.84	34.19	0.086	8.073	1.090	18.842
2	98.7	19.16	35.15	0.084	8.252	1.088	19.164
0 = control, 1 = 0.16 g Si/kg soi, 2 = 0.32 g Si/kg soil							

Table S13 Fecundity of BPH feeding on rice plants

treatment	eggs/female	treatment	eggs/female	treatment	eggs/female
0	112	1	96	2	78
0	192	1	40	2	92
0	183	1	136	2	99
0	136	1	99	2	96
0	86	1	144	2	103
0	99	1	78	2	30
0	121	1	131	2	143
0	140	1	89	2	79
0	141	1	124	2	138
0	143	1	169	2	134
0	104	1	90	2	131
0	64	1	94	2	105
0	176	1	87	2	86
0	193	1	101	2	104
0	131	1	131	2	86
0	108	1	133	2	85
0	133	1	63	2	142
0	162	1	131	2	83
0	123	1	80	2	81
0	170	1	48	2	58
0	246	1	139	2	64
0	73	1	121	2	144
0	176	1	80	2	89
0	158	1	90	2	106
0	236	1	123	2	109
0	201	1	109	2	101
0	128	1	115	2	118
0	189	1	143	2	94
0	121	1	125	2	89
0	184	1	156	2	93
0 = control, 1 = 0.16 g Si/kg soi, 2 = 0.32 g Si/kg soil					

Table S14 Nymphal survival of BPH feeding on rice plants

treatment	Nymphal survival rate (%)
0	52.86
0	54.29
0	54.93
1	45.21
1	47.95
1	48.65
2	47.22
2	45.83
2	47.95
0 = control, 1 = 0.16 g Si/kg soi, 2 = 0.32 g Si/kg soil	
